# Supplementary material for: In-Vitro MPI-guided IVOCT catheter tracking in real time for motion artifact compensation
Source: PLoS One. 2020 Mar 31;15(3):e0230821. doi: 10.1371/journal.pone.0230821 (PMC7108728; doi:10.1371/journal.pone.0230821)
Supplement: Attachment — Submitted filename: Rebuttal__Tracking_IVOCT_catheter.pdf [file pone.0230821.s001.pdf]

IN-VITRO MPI-GUIDED IVOCT CATHETER TRACKING IN REAL TIME FOR MOTION ARTIFACT COMPENSATION

FLORIAN GRIESE, SARAH LATUS, MATTHIAS SCHLÜTER, MATTHIAS GRAESER, MATTHIAS LUTZ, ALEXANDER SCHLAEFER, TOBIAS KNOPP

1. General Comments to the Reviewer and the Editor

We would like to thank the reviewer and the editors for their helpful comments and we have further improved the manuscript accordingly. Please find our point-by-point response below.

* 1. Please ensure that your manuscript meets PLOS ONE's style requirements, including those for file naming. `http://www.journals.plos.org/plosone/s/file?id=wjVg/PLOSOne_formatting_sample_main_body.pdf` and `http://www.journals.plos.org/plosone/s/file?id=ba62/PLOSOne_formatting_sample_title_authors_affiliations.pdf`
Answer: We have adapted the manusscript according to the PLOS ONE's style requirements. The file naming is changed.

* 2. In your methods section, please provide more information about the provenance of the dataset, and clarify whether the study involved human subjects.
Answer: We added a sentence regarding the provenance of the dataset and state that this study is an *in-vitro* study and it does not involve human subjects.
Revised: L198: "The described phantom experiments are performed *in-vitro* and do not involve human subjects. The datasets are acquired with the described imaging devices (pre-clinical MPI scanner, spectral-domain OCT system). The MPI data processing is implemented in Julia, while the OCT data processing is written in Matlab."

2. Answers to Reviewer 1

* l22: Please choose: "proximally rotated catheters" or "rotated proximal catherters" depending on what your want to express.
Answer: Thank you. The catheters are rotated proximally, we revised the wording accordingly.

* l74: You are claiming the lacquer does not affect the OCT beam profile. Please either give a suitable reference or show your measurements supporting this claim.
Answer: Thank you for this helpful remark! We did not identify any difference between OCT images acquired with lacquer marked catheter and normal catheters. Therefore, we weakened our statement.

Revised: L74: "The OCT images acquired with the marked catheter provide suitable image quality, whereas the phantom structures are still apparent for later segmentation algorithms."

* l92: then − > than.
Answer: Corrected. In the description of our catheter profiles (section 2.4), we switched to a different wording.

* l97: Either "contains" or "consists of" but not "contains of".
Answer: Corrected.

l136: What is your criterium for declaring a value an "outlier"?
Answer: A Ransac algorithm is used to eliminate the outliers. It randomly takes a subset of points and calculates the regression line. With these initial model parameters the entire data set is tested for consistency. A data element is declared as outlier when it does not fit the model with a certain threshold. The fitting elements are selected as inliers. This process is repeated with more randomly selected subsets to obtain a data set with inliers only. Additionally, extreme outliers are excluded via thresholding as they differ from the expected value by several millimeter. In these cases, the images are affected by heavy noise and the localization algorithm falsely detects a high intensity noise voxel as marker position.
Revised: L137: "Additionally, outliers are removed with a Ransac algorithm and extreme outliers are excluded via thresholding. In these extreme cases, the images are affected by noise and the localization algorithm falsely detects a high intensity noise voxel as a marker position. Further, the trajectories are smoothed to ensure a continuous trajectory."

* l184: While mathematically correct, please avoid speaking of "decreased" in this context, rather use "set". A decrease leading to a higher absolute velocity if counter-intuitive for the reader.
Answer: Thank you for this helpful comment. We have corrected it and use "set" in section 2.4.

* fig4+6: I recommend to combine these two figures only displaying the contents of 4b and 6a. A constant velocity (4a) needs no depiction and I do not see the benefit of having the velocities graphically presented in 6b.
Answer: Thank you for tighten the content of our paper. We merged Figure 4 and 6 to one Figure and used 4b and 6a only.

* l210: You are stating that the tracked positions are in good agreement with expected values from 18mm to 10mm. Looking at the error bars of your measured values I would rather cut this argument short at 11mm.
Answer: Thank you for this commit! We agree with your observation and changed the range to 18mm to 11mm accordingly.

* fig7 (caption): Here you state the good agreement was from 18mm down to 6mm. This is (a) in contradiction to your own statement in the text (18mm-10mm) and also please consider my previous comment.
Answer: Thank you. We corrected this mistake. As in the comment before we changed the range to 18mm-11mm.

* l215: The values of AE and RE given here do not agree with the ones given in tab1. Please verify!
Answer: We corrected this disagreement. The values from the Table are the correct values.

* l218: You are addressing "a small deviation" of the measured values from the expected ones. Looking at fig7b the measurements seems not to capture the first change in velocity at all since it starts at a wrong slope. Please discuss this result in more detail! Are there issues with higher velocities?
Answer: Thank you this precise observation! You are right, the first change of the velocity is not reflected by the values from the measurement. One explanation could be that the first velocity change is close to the border of the MPI FoV. The voxel intensities representing the marked catheter tip are shaped as "circle" in the MPI images (see Fig.2). If this "circle" has no entered the image completely, the center of mass localization algorithm might misinterpret the position. We updated the caption in Figure 6 and added this aspect in the discussion.
Revised: Caption Figure 6: "MPI measurements for BA profile: The first change of velocity is not captured by the measurement. After the first change the measured distances in x over time are in agreement with the different velocity v_0 and v_1 with only slight deviations."
Discussion L302: "For the BA profile the first change of velocity could not be captured by the measured values. One reason could be that the velocity change is close to the border of the FoV. The voxel intensities representing the marked catheter tip have circle shaped form in the MPI images. If this circle shape has not entered the FoV completely, the center of mass localization algorithm might misinterpret the position."

* l227 and fig8: Similarly to the above case the velocity seems to be underestimated while you are constating a good accordance. Please comment on the too small slopes for higher velocities.
Answer: Thank you for this comment. The caption under the figure is misleading. We have changed the caption accordingly. In the discussion we try to explain this underestimation.
Revised: In Text L232 :"For the HBA profile the distances in x over time between 18 mm and 6 mm are shown in Fig. 7a. Overall, the tracked x positions resemble the movement of the catheter being pulled back and forth. The turning points between velocities $-v_0$, v_0 and again $-v_0$ can be clearly identified. However, in the first two segments the velocity is underestimated as the tracked x positions are not in full accordance with the expected x positions (red). In the third segments the tracked x positions agree with the expected values."
Caption Figure 7a: "The measured distances in x agree with the set catheter movement. The turning points are clearly visible. The velocity, however, is underestimated in the first two segments. In the third segment the measured distances in x agree with the expected positions in x."
In Discussion L307: In addition, in case of the HBA profile deviations occur around the turning points seen in Fig. 7a), which lead to an underestimation of the velocity. These deviations can be linked to the catheter setup with a proximal actuator such that the pullback is increased by the shrinkage and stretching of the flexible catheter. It is also worth noting that the back and forth motion of the catheter is also visible in the y- and z-positions seen in Fig. 7b-c) as the vessel phantom is not placed perfectly in accordance with the x-axis.

* l236/7: Three times the units (mm) are missing.
Answer: Corrected.

* l239/40 and tab3: The values given for AE/RE do not agree between table and text.
Answer: Corrected. The value in the table are the corrected values for AE/RE.
